# Is it Still Ok to be Ok? Mental Health Labels as a Campus Technology

**DOI:** 10.1007/s11013-023-09819-3

**Published:** 2023-03-24

**Authors:** Neil Armstrong, Laura Beswick, Marta Ortega Vega

**Affiliations:** 1 Harris Manchester College, University of Oxford, Oxford, OX1 3TD UK; 2 Kings College, London, UK

**Keywords:** Mental health, Stigma, Labels, Biomedicalization

## Abstract

This article uses ethnography and coproduced ethnography to investigate mental health labels amongst university students in the UK. We find that although labels can still be a source of stigma, they are also both necessary and useful. Students use labels as ‘campus technologies’ to achieve various ends. This includes interaction with academics and administrators, but labels can do more than make student distress bureaucratically legible. Mental health labels extend across the whole student social world, as a pliable means of negotiating social interaction, as a tool of self-discovery, and through the ‘soft-boy’ online archetype, they can be a means of promoting sexual capital and of finessing romantic encounters. Labels emerge as flexible, fluid and contextual. We thus follow Eli Clare in attending to the varying degrees of sincerity, authenticity and pragmatism in dealing with labels. Our findings give pause to two sets of enquiry that are sometimes seen as opposed. Quantitative mental health research relies on what appear to be questionable assumptions about labels embedded in questionnaires. But concerns about the dialogical power of labels to medicalise students also appears undermined.

## Introduction

Brian is taut, tense, and watchful. His gentle self-mockery speaks to a degree of self-awareness that at times blends into an uncomfortable self-consciousness. He is both a thinker and an over thinker. When he talks about his life it sounds dramatic, almost mythic: adventures, quests, ordeals, victories and losses against foes. He excels in his academic work, and fits an active social life into the relatively small container of his spare time. Planning comes naturally to Brian. Making plans, and if necessary, exerting his will to follow through on those plans is his default approach. It is how he sets about negotiating life’s difficulties, how he contains his dramas, orients his energies, constructs his narratives. As a graduate student in the social sciences, who has taken courses in critical mental health, Brian might be seen as a highly informed consumer of mental health labels. He told me he regards labels as a kind of intellectual property used by the representatives of global Biomedicine to market products and services. But he also recognises the positive value of labels in framing research and developing interventions. He went on to say that for years he has considered herself to have manic depression or bipolar disorder, but that he recently had a shock when assessed by the university disability service:

‘People told me I was bipolar, that my days were a succession of highs and lows. I was never formally diagnosed, and I knew I wasn’t a perfect fit, but I read up on it, it helped, it described me, I could see that this was who I am. But now, now I’m not so sure.’

If he doesn’t have bipolar, he is not entitled to access the disability service. But Brian made it clear that is not the main issue. Instead, he said that he uses the label bipolar and the categories around it to make sense of his life. This means he thinks of himself as having periods of ‘high mood’ and ‘low mood,’ and sometimes a combination of the two, painful ‘mixed moods.’ Some activities—partying, for example—might seem risky, having the potential to precipitate a ‘mood episode’ but also be constitutive of a ‘mood episode.’ Brian said he finds these medical categories to be a useful way of identifying vulnerabilities that he needs to plan for. Thinking of his life in these terms also means that events that might attract disapproval, or be read as unattractive, or weird, or unlikable, may instead be interpreted as part of a dark, even romantic health condition. The label bipolar is a kind of social absolution, an insulation against social failure. It also creates relationships. Bipolar labelling suggests to Brian that his low mood is the same low mood that drove Robert Lowell’s poetic genius. He even jokes: ‘It may be my only way of being close to genius.’

As we talk, I see that removing a label can be a complex issue. If Brian doesn’t have bipolar, what is he to make of his life? Removing the label is like pulling a hermeneutical rug from beneath his feet. He may not have a mood disorder, but he still feels in some sense disordered. His intense inner life and picaresque trajectory have the flavour of bipolar, at least in the popular imagination, and seem unlikely to change. Losing the label means he has to reinterpret his past and find new ways to negotiate his future. He will have fewer resources to deal with a competitive social world. I could see that Brian’s relationship with the mental health label bipolar was complex, and disentangling himself might well prove difficult and emotionally costly.

Most influential publications in the social science of mental health explore labels as a source of harm. In Goffman’s classic phrasing, mental health labels ‘discredit’ the labelled, ‘spoiling’ their identity (Goffman 1969). Goffman described various ‘information control’ strategies used by labelled individuals to minimise stigma, with the goal of either ‘passing’ as normal, or at least reducing one’s visibility on the social stage. For Schiff, labels have a paradoxical, self-fulfilling quality, in that they deepen the problems they purport to describe (Scheff 1966). These key early texts have generated a rich literature, embedding foundational concepts and establishing a particular direction of interpretive travel. Estroff confirms Schiff’s work ethnographically, showing how long term mental health patients become caught in a self perpetuating cycle (Estroff 1981). Thornicroft describes how stigma radiates out, impacting, on housing, employment, intimate and social relationships, self image and sense of self worth and family life (Thornicroft 2006). Using ethnographic material from a women’s homeless centre in Chicago, Luhrmann sees how labels contribute to the ‘countless small humiliations’ experienced each day by users, sometimes leading to ‘social defeat’ (Luhrmann 2007:159). Jenkins and Carpenter-Song describe ten strategies deployed by people who have recovered from the acute stages of schizophrenia in the US but still find themselves ‘saturated’ by stigma (Jenkins and Carpenter-Song 2008). They note ‘These strategies highlight the fact that persons with mental illness can be not only exceedingly socially aware but also strategically skilled in response to social assaults on their personhood and survival’ (ibid: 404). Similarly, Baines describes how individuals with autism carefully distance themselves from their diagnosis, to limit its impact on their lives (Baines 2012).

Our ethnographic work suggests that things might be changing. In the contemporary university campus in the UK, labels of all kinds are ever present. Mental health labels have become part of the cultural landscape. The students we worked with emerged as self-consciously strategic, sometimes ironic, at other times playful, in relation to mental health labels. Brian, for example, finds bipolar to be a kind of genre that he uses to narrate his life history, a set of interrelated ideas that help him structure his day to day planning, and a fertile topic of self-deprecating humour. He is far from unusual in not primarily being interested in minimising unwanted reputational damage, or trying to ‘pass’ or distance himself from labels. Brian explained that he thinks that bipolar defends him from reputational damage, and that losing it feels exposing, as if a protective layer has been peeled off. Many students told us about how they harness the useful social effects of labels. Medical terms may still discredit an individual or spoil their identity, but they also make a person legible, and produce a kind of social power in engagements with institutions, in social settings, and in the formation of a sense of self. Especially online, labels can even promote personal attractiveness or sexual capital. Mental health categories emerge as a kind of resource. We call them ‘campus technologies.’ Looked at from this point of view, those who remain unlabelled might be free of some of the harm of mental health stigma, but they might also be relatively less equipped when compared to their labelled peers. In a campus inundated with efficacious labels, ‘being ok’ might put a person at a disadvantage.

Our larger argument is that the social life of mental health labels no longer conforms to the assumptions that underly a great deal of academic work. Janis Jenkins has described how the struggle for mental health ‘is not just against an illness and its symptoms but also for a normal life’ (Jenkins 2015:261). We suggest that this orientation towards ‘normality’ can no longer be assumed. ‘Normality,’ at least if it consists in being label-free, is not what it used to be. Seeing labels as campus technologies may be disruptive to two sets of enquiries in particular. First, our work raises methodological questions. Mainstream social science research that is based on questionnaires is reliant on labels having fixed, interpretable, unambivalent meaning. Researchers assume that they know what a student means when she describes herself on a questionnaire as ‘depressed,’ for example. Our research suggests that ‘depressed’ can mean a wide range of things, depending on context. The meaning of mental health labels is not tied down by medical authority and can be appropriated and extended by students in ways neither intended nor anticipated by researchers.The interpretation of responses to questionnaires is thus unsettled and can’t be assumed without further enquiry. But equally, our analysis pulls against the stream of critics working from a Foucauldian perspective, who see labels are a part of an apparatus of subjectification, vectors of biopower that internalise the moral project of the state (e.g., Rose 1998). In such accounts, labels become instruments of discipline, even domination. There is little scope for agency other than resistance. But the students we worked with appear to have much more room for manoeuvre. Rather like the mindfulness practitioners described by Joanna Cook, who cannot simply be dismissed as neoliberal dupes, the students we worked with are aware of the problems of biomedical terms and have appropriated medical knowledge in such a way that labels no longer seem to have the capacity to be oppressive or hegemonic (Cook 2016).

Our work is part of the UKRI-funded Student Mental Health Research Network, ES/S00324X, a four-year funded network based at King’s College London that seeks to formulate a clearer narrative about the prevalence and causes of mental illness among students, and the effectiveness of interventions from mental healthcare. The aim of our sub-project was to try to get a richer sense of how mental health labels play out in the lives of students. Initially, the plan was to coproduce with students, using ethnographic methods to develop a thick, contextual description of mental health labels. Coproduction can mean many things, including collaborative and relational working in research design and methods, in representing the results of research and in the construction of analytic concepts (Boyer and Marcus 2020:1). It often introduces a further layer of openness or indeterminacy into the already flexible anthropological research process. From the start we intended to work in an open, exploratory and egalitarian way. Unexpectedly, this led to new directions even before starting formal ‘fieldwork.’

Our sub-project began in December 2018 with a series of training sessions in ethnographic research given by Armstrong to a group of students, including Beswick and Ortega Vega. The students had responded to a series of adverts located online. The response rate was high, and our selection process included attempts to make the group demographically diverse. None had a background in anthropology. During training, it emerged that there was an apparently poor fit between the anthropological literature on mental health labelling and the student ethnographers’ experiences and impressions. As we chatted it became clear that mental health labels have positive features. For the students, labels are not fixed or inert, but act as a tool that might be actively engaged with, and even used strategically. This seemed obvious to the students, even though there was limited reference in the literature. We received ethics clearance in February 2019, after which we used posters placed in universities to recruit interlocutors. Interviews were held up until September 2020. They were recorded and transcribed, according to our ethics approval (HR-18/19-8671). From October 2020–December 2021 the three writers met weekly online to discuss our data and to write together.

Boyer and Marcus write about ‘the revelry of collaborative anthropology’ stressing the creative and even transgressive potential of this way of working (ibid:18). This was our experience too. It had at least three aspects. First, we found there to be no clear dividing line between ostensibly pedagogic training sessions, the findings from research interviews, and discussion during writing. Our research process began not in ‘the field’ but during training and it continued in a series of impromptu follow-on conversations. This resulted in a shift in the focus of interviews. In other words, collaborative working breached boundaries between planning, data collection and analysis. Ethnographic insight emerged throughout the process. We felt we were all learning all the time. Second, the original plan was to analyse interviews in the light of anthropological theory and wider interdisciplinary literature. But as the writers discussed the data, our own experiences came to mind. The lived experience of the three writers thus entered our analysis, such that all three authors have a double role as interlocutor and author. This means 'students' may refer both to researcher and interlocutor, interviewer and interviewee. For reasons of confidentiality, we do not here explicitly differentiate between autoethnographic material and material drawn from interviews. Thirdly, engaging in coproduction also brought a heightened sensitivity to the collaborative nature of all knowledge production. Discussions amongst the writers led to a shared sense of how our ideas and experiences develop through informal interactions with others. Working like this means that demarcating the frontiers of who might be construed an ‘informant’ is problematic. All individuals we give names to, and everyone we directly quote, are either members of the writing group, or students who have consented to join the study as interviewees. But there is a huge, unstated and ill defined hinterland of informal interlocutors who have indirectly contributed to this paper. We do not assume this pool to be representative, but we suggest that it is large enough for us to sustain our argument.

Oliver and colleagues commented on what they call ‘the dark side of coproduction’, suggesting that the costs may outweigh the benefits and warning ‘it takes investment, skills, time and courtesy’ (Oliver et al. 2019:7). Williams and colleagues are more optimistic, stressing the potential of coproduction and suggesting that much of the supposed dark side is really bad practice (Williams et al. 2020). Like Williams and colleagues, our experience has been that the challenges of the method are also its strengths. We have developed shared expertise in careful, attentive, (and we hope, courteous) discussion and coworking. Unlike Oliver and colleagues, we see the flexibility and relative lack of specification to be an asset in at least this instance. By not following defined strategies and protocols, we believe we were able to respond more sensitively to the social world we were engaging with (Armstrong and Agulnik 2020:891). In this paper, a principal benefit is a kind of boldness. The case we are making goes against trends in the literature. But we found widespread recognition amongst our interlocutors, and coproducing ethnography appears to be a way of transferring that confidence from data gathering into the process of writing.

The paper starts out by exploring ethnographic material on mental health labels as part of the conceptual apparatus of the university. We show how the institutional legibility of labels does not fix or exhaust their meaning. Instead, students creatively, strategically and sometimes humorously adopt and rework labels, such that labelling becomes a familiar way of validating experience and negotiating day to day social encounters. The expressive resources provided by mental health labels make them helpful to students. We draw on the work of Eli Clare to better understand varied relationships a person might have with labels (Clare 2017). Labels might sometimes stigmatise, but they also offer the potential for self exploration, sociality, and humour. This is particularly the case in online dating, where labels can be deployed to contribute to a person's desirability. We go on to consider the consequences of our findings for questionnaire-based research, and think through some wider implications.

## The Social Life of Labels

A mental health label can be years in the making, representing the successful outcome of extensive, uncomfortable and sometimes bruising encounters with medical professionals. But, we were told, a label can be worth it because it helps people make sense of experiences, and validates distress. As a result, some of our interlocutors invested a great deal of trust in labels, and became experts in the associated medical guidance. For members of this group, any advice or information on self-management created by organisations or individuals outside the medical profession was treated with caution. Likewise, advice, jokes or comments which were seen to belittle the illness or grossly misunderstand the perspective of somebody with lived experience, were heavily criticised. But this is just one particular relationship between a student and mental health labels. Students like this, with what we may describe as relatively orthodox positions regarding labels, are perhaps particularly likely to participate in research investigating student mental health, but their experiences cannot be taken as representative of the wider student population.

Sarah Crook suggests that reports of a crisis in student mental health (and related concerns with neoliberalism and the supposed ‘snowflake generation’) are exaggerated (Crook 2020). She remarks: ‘the health services at contemporary universities are part of the story’ because they make student mental health ‘legible’ (ibid:219). For Crook, this process of ‘making legible’ means that student distress is turned into something apprehensible to university institutions. If distress is labelled as mental health, it may be documented, measured and planned for. Our student interlocutors are well aware of this. They repeatedly stressed that the world they live in continuously asks them to label themselves. It can seem incessant. University institutions ask students to label themselves in various ways: health, disability, age, gender, ethnicity, sexual orientation, family educational background, among others. Whether it’s a request for mitigating circumstances, or even to join a university society, labels are everywhere. This suggests that mental health labels can’t simply be regarded as medical terms derived from research or clinical practice, with fixed meanings and properties delineated by medical authority. As Leonard J Davis puts it: ‘health is something that belongs to the promoters of an explanation as well as those who oppose that explanation (Davis 2010:130). And health labels travel far from clinical settings. They are what Bowker and Starr call ‘boundary objects’ in that they cross institutional frontiers and ‘inhabit several communities of practice and satisfy the informational requirements of each of them.’ (Bowker and Starr 1999:297).

Our attention here is directed towards ways that students represent their distress, and how those representations play out in other spheres. We do not suggest that that the mental health labels used by students are fraudulent or false. The distress and disability that lie behind labels can be painfully real. Neither do we argue that students are relabelling experiences of everyday distress that previous generations of students did not seek to medicalise. But we feel we can’t ignore the social impact of labels and the expertise of the labelled in recognising and strategically deploying the power of labels. A mental health label may function in a range of settings, from housing, access to support, leniency in exams, or diversity and inclusion monitoring. However, the way they function, and the meaning they hold, may subtly shift. Again, the students we worked with demonstrated they are aware of this. Anxiety cited during university disciplinary hearings may be rather different from anxiety discussed in a Whatsapp group of anxious friends. This generates a kind of expertise. Students are no longer simply labelled, but should be seen as active and sometimes skillful labellers in their own right.

Students described to us how to use medicalised terms in conversations with faculty and university staff in such a way as to pick out “key” warning signs. Requests for mitigating circumstances regarding extending coursework deadlines are not accepted unless supported by an official diagnosis from their GP or a healthcare professional. Non-medical terms like ‘burned out’ or ‘stressed’ carry little weight when engaging with the university bureaucracy. In Fassin and Rechtman’s terms, medical labels have a different ‘moral economy’ (Fassin and Rechtman 2009). It can be the cause of some irritation:

The fact that you can’t approach them saying ‘I’m having a really bad time I need support,’ the fact that you need to evidence it with mental health professionals makes it so hard to access it in a timely manner.

The students we worked with found themselves in an environment that imposes labels. University bureaucracy is responsive to certain kinds of label, and students have consequently adapted. Facing long waiting lists and extensive paperwork to access support, students have learned to conform. Vinh-Kim Nguyen and colleagues developed the notion of ‘therapeutic citizenship’ to account for the extremely high levels of adherence to antiretroviral therapy among HIV positive people in francophone Africa (Nguyen et al. 2007). They found that individuals who received the HIV diagnosis took it as an ethical project, a way of remaking themselves, their rights and obligations. The arrival of the label of HIV, and the technologies of care that accompanied it, transformed social relationships. There appears to be some overlap with our material. But the students we worked with are therapeutic citizens only intermittently and often in a rather qualified way. Some were wholehearted in their embrace of labels but others were reluctant, using labels whilst regarding them as slightly compromising, and not wholly authentic to their experience.

Our interlocutors frequently reflected on the power of diagnosis to validate experience. The words “anxiety” or “depression” hold much more authority than describing fatigue, difficulties concentrating, and other associated experiences:

“I remember the first time I submitted a mitigating circumstances form, you know it’s like you’re laying everything bare to them and it’s almost demeaning in a way. It’s like I have to explain why I’ve been depressed, I have to explain when I started taking medication, I have to explain the entire history of my mental health and justify why I need an extension for an assignment. I feel like it’s not the nicest help, it’s not the nicest way you can help people.”

The right mental health labels provide an advantage that non-medical terms or emotion-focused language would not. Our student interlocutors frequently told us that they feel that if they did not adopt labels their personal experiences of distress would be invalidated or somehow not count.

As members of a community that seems to constantly be at heightened stress, overburdened by pressures connected to workload and social life, mental health labels help students to be taken seriously by their peers and generate a more sympathetic response. Amy Chandler found that whilst people who self-injure have multiple and richly varied understandings of self-injury as a sometimes helpful activity, reductive and stigmatising mental health labels inhibit help-seeking (Chandler 2014, 2016). In contrast, we found that in many instances, mental health labels were seen as a means of sharing experiences, and deflecting potential claims to inauthenticity or being attention-seeking. The students we worked with who identified as having eating disorders seemed particularly aware of these features. In one conversation, we discussed reasons why students sometimes prefer to use diagnostic language when describing mental health experiences:

“I think they probably mostly talk in terms of diagnosis because you don’t want to feel like you’re attention seeking I guess, so having a diagnosis kind of validates it if you know what I mean.”

She cited experiences with her eating disorder where before receiving a diagnosis, her parents were sceptical and even hostile, saying: “I think you just like the attention this is getting you”. For her, using a diagnostic label provides a sense of validity or realism to painful but contested experiences. This is another example of labels making individuals less isolated, and providing a means of at least attempting to overcome stigma or unwanted disapprobation.

Some labels are more palatable than others. Brian, who we described at the start of this paper, found bipolar to have a certain romance. We found that depression and anxiety are thought by some to indicate depth, and can help connect with others. In contrast, psychosis is liable to alienate or alarm. But labels don’t operate the same way or have the same meaning in all groups. A mental health diagnosis might be more favourably received amongst humanities students, the dark glamour of suffering indicating insight, depth and sensitivity, whilst, in contrast, medical students appear to be cautious about mental health labels. We were told that they were reluctant to talk to faculty members about distress out of fear of being seen as not fit to practice. Medical students also felt judged by their peers and lecturers if they showed distress:

“When it comes to something more organic, is that the right word? Like a “medical” mental health issue, like psychosis, it’s sort of the impression that lecturers are like “Yikes, can’t deal with this! Maybe talk to your GP”. It’s really scary for us because these are, you know, distinguished people, professors, people with PhDs and all that, but I guess it really just comes down to the fact that people still intrinsically are just scared or don’t know how to approach this.”

This echoes findings in biomedical research literature about high rates of stigmatising attitudes and behaviours amongst mental health professionals (e.g., Horsfall et al. 2010). One student shared the fear of labels impacting their ability to engage with their medical degree:

“I think many of my friends see seeking support outside the faculty as slightly better in terms of thinking ‘oh it might ruin my career!’. If students think “They’re going to think I’m crazy, they might kick me off the course” they might not want to seek help internally and worry that their course leads would decide that they need time out or they can’t do this course anymore.”

Medical students frame mental health labels in terms of functional impairment: their ability (or lack of ability) to keep up with work, where their peers in other disciplines are more likely to consider mental health as an emotional experience, even a tragic but romantic character flaw (Ortega Vega 2021).

Expertise in labelling had unexpected features. We discovered that mental health labels are sometimes deployed as a way to prevent confrontation or deflect further scrutiny. We heard of instances where students, whether or not they identify as having mental illness, use labels such as ‘anxiety’, ‘depression’ and other common mental illness labels to explain or even justify unrelated behaviours. They explained that it is a lot easier to tell a friend that you can’t see them because you are struggling with mental health, rather than telling them it is because you don’t want to go out. One student shared some discomfort in engaging in this behaviour:

“It’s obviously not a bad thing that mental health is normalised and people are sympathetic towards struggling individuals, but there’s kind of that worrying underlying sense that this normalisation means that it’s not something that is being treated as seriously as it should be. We use humour as a coping mechanism, because if you tell someone that you’re doing really badly, more often than not people are gonna be like “Oh yes. Mood. Same” and you laugh between yourselves because humour is such a coping mechanism for youth. It’s not funny sometimes but you just have to engage in it as part of the conversation.”

In this case, mental health labels protect against, rather than cause, social ostracization. We were told that if a group of friends are experiencing anxiety due to coursework, being the ‘lucky one’ who is finding things less distressing can create a disconnect to the group. Being ok can be socially risky.

Part of the complexity of mental health labels lies in what is not being said. The prevalence of a certain kind of talk about vulnerability doesn’t necessarily license the disclosure of all forms of vulnerability. As Emily Martin put it in her ethnography of bipolar disorder in the US, labels ‘allow people to keep their interior landscape closed to comparison…[and so] act as a shield against revealing more intimate experiences’ (Martin 2009:141). The students we worked with appeared more ready to talk about their mental health diagnosis or talk of anxiety than feelings of loneliness or about deeper insecurities and struggles. We were told that it feels easier to tell a friend or personal tutor that you have been experiencing anxiety, than describing experiences of struggling with feeling lonely, homesick, or stressed. In this context, students may not be internalizing the label or truly identifying as someone with mental illness. Rather they are using a label that means they don’t need to disclosure more sensitive, potentially more stigmatising personal information. With both academic staff and friends and acquaintances, labelling is as much a technique of concealment as it is exposure, providing safety against a more difficult conversation. An interviewee, who had a long career in a University Counselling service confirmed this finding. He expressed concern regarding students’ inability to talk to one another about their homesickness and loneliness. He said that opening up about mental health is often a way of shutting down. He put it like this: ‘as soon as someone shuts themself off as ill, it closes down the conversation. I want staff to be able to lean into the hard questions and ask what’s really going on for students. We’ve created a culture where people are terrified to ask anything that smacks of emotion’. It seems that mental health labels offer an opportunity to be vulnerable without being vulnerable.

## ‘I Knew Just How Honest to be:’ Labels and Tone

Seriousness is not a human default. Students like to joke, and value playfulness and irony, but there is no consensus about when humour or exaggeration regarding mental health labels is appropriate. We encountered individuals who perceive the ridiculing or exaggeration of mental illness as undermining their medical condition and the difficult experience attached to it. This is still the case even if the jokes or anecdotes are made by someone who experiences the same illness. For example, somebody with bipolar disorder might play on their diagnosis to avoid repercussions for inappropriate behaviour. One student told us: ‘I’m overdrawn, but if I tell the bank I was manic, they’ll have to let me off’. Such trivialising and strategic, perhaps rather cynical use of medical diagnoses was a subject of frustration for others, who presented their own mental health label as a signifier of a turbulent and difficult personal road travelled, and so something to be treated with integrity and respect.

Throughout our research we noticed subtle shifts in how our student interlocutors spoke about mental health labels. They adopted different registers that seemed critical to meaning but hard to pin down. Sometimes they were joking, or half joking; at other times they were slightly ironic, or dismissive, or playful. In response to one question, a student might be cryptic, perhaps a touch disingenuous. But in response to a second seemingly synonymous question, the same student might be forceful, making affectively charged normative statements. What was said often seemed less important than how it was said. As Garkinkel argues in his classic paper on ethnomethodology, what people are talking about can be difficult to disentangle from how they talk about it (Garfinkel 2021 [1967]:28). One of the methodological challenges we faced was how to adequately capture these shifts in tone.

Tone has not been the focus of much analysis of labels. Some of the more elaborated theory describing the interaction of people with biomedical labels, such as Ian Hacking’s notion of ‘looping’ or Paul Rabinow’s concept of ‘biosociality’ do not consider tone (Hacking 1995; Rabinow 1996). Nonetheless, we find tone to be central to the social life of mental health labels. The openness of ethnographic attention lends itself to an awareness of tone, and it is no surprise that humour, for example, has received attention, even if intermittent, from some major figures in the history of anthropology (Radcliffe-Brown 1940; Douglas 1975, 1996). Andrew Sanchez’ work on profane—and even racist—joking on a factory floor in central India argues that joking is socially helpful because it lessens inter-ethnic tensions: ‘The irony of offensive jokes is that their profane sociality is able to undermine intolerance.’ (Sanchez 2016:309). But much of our ethnographic material reveals less clear-cut tonal shifts. This is a more extensive phenomenon than is theorised by Paolo Heywood’s account of double morality (Heywood 2018). Mental health labels are sometimes a topic of humour, sometimes completely serious, sometimes the subject of light mockery, or exasperation, or smiling impatience. In a study on the stigma around weight and fatness in the US and Japan, Cindi SturzStreetharan and colleagues note that whilst people may often be polite about weight in public, when they feel less exposed, they may express strongly stigmatising views about body size (SturzStreetharan et al. 2021:4). This means attending to tone is critical: ‘Our argument is that these verbal acts of politeness [in public] effectively *deepen* stigma, in part because the underlying attitudes that see fatness as abhorrent are not disrupted or challenged’ (ibid: 5).

Dark humour in social bonding and, more specifically, mental illness related comedy is an emerging area described in the psychology literature. Braniecka and colleagues conducted a study where they found that humour is a meaningful way to regulate our emotion, particularly those dealing with depression or mental health issues (Braniecka et al. 2019). The results from the study found that using humour was associated with an increase with positive emotions and a decrease with negative emotions. In addition, the study found that dark humour helped people to distance themselves from difficulties and hardship. This has been previously linked by students to the media and meme culture, where dark humour, particularly to cope with mental health experiences, is prevalent. This humorous tone is incongruous with the seriousness of the label, although it has been described as a mechanism to create social bonding over a negative experience which may reduce loneliness and low-self esteem (Overholser 1992). Self-reflective internet memes, particularly “dark memes'' that are self-deprecating in nature, provide a platform for students to communicate about mental states using a shared language that allows them to cope with negative emotions by laughing at their struggles (Tariko and Anasih, 2019). This sense of connection or mutual understanding through humorous medium may encourage students to use mental health labels to feel a sense of belonging.

One way forward might be to consider first-person accounts of labelling. Disabled and genderqueer writer Eli Clare has experience of a range of different labels. He writes against the grain of contemporary biomedicine, reflecting on a lifetime evaluating, contesting, rejecting, renegotiating, even repurposing labels. He explains:

My relationship with mental retardation, cerebral palsy, schizophrenia, and gender identity disorder (GID) range widely. The first of these diagnoses has fallen by the wayside, even if it still stalks me in the form of hate speech. The second found me during my parent’s search for a cure, and is a convenient shorthand when I request disability access, navigate the medical-industrial complex, or deal with random curiosity, but it has never orchestrated a life-changing revelation. The third I narrowly escaped, grateful not because seeing visions and hearing voices are inherently bad or wrong, even when they create havoc, but because the medical treatment and social conditions accompanying that diagnosis are often dreadful. But the fourth I actively sought out (Clare 2017:139)

Clare regards all four terms as risky and unsatisfactory. ‘Mental retardation’ is (clearly) a wounding medical term that was applied to Clare in his childhood, has now been abandoned but, it seems, can never quite be shaken off. The diagnosis of schizophrenia he feels fortunate to have evaded not primarily because it doesn’t describe him very well (although it doesn’t), but because the social effects of the label and the medical care it invokes are so undesirable.

Clare actively uses the final two labels. He sometimes, albeit with reluctance, describes himself as having cerebral palsy because it works as a shorthand or heuristic, a way, however imperfect, of communicating with people and with institutions. Clare emerges as a kind of dissident with regard to the term, mindful of its limitations and potential harms and painfully aware that being forced to use it reflects his limited freedom to define himself. GID is rather different. Clare has accessed various medical interventions for gender transitioning and knew that accepting and using the category GID was a necessary part of the process. He suggests that we should think of GID not as a static category but as a ‘tool embedded in time, space, culture and science’ (ibid:140). In our terms, GID was, for Clare, a bureaucratic technology.

Tellingly, in describing how he passed through the various preliminaries to surgery, Clare remarks, archly: ‘I knew just how honest to be’ (ibid:141). The reader is not left doubting the sincerity of Clare regarding transitioning. But, it appears, the social world in which he finds himself demands a certain pragmatism, forcing him into stances falling somewhere between total honesty and outright dishonesty, subtle tonal positionings that we see also in our ethnographic material. Clare even finds a continuity between mental health categories and queer labels. At first sight this might appear incongruous and unlikely. But in Clare’s careful account, labels are technologies of self-creation and self-discovery. And our student interlocutors make the same moves. In their accounts, heterogeneous categories jostle and bump together. Some (eg ‘manic’, ‘psychotic)’ are linked to biomedicine and refer to perceived dysfunctions or pathologies, others (‘nonbinary,’ ‘gay’) identifying patterns of desire or sexual identity.

Progressive voices in mental health see labelling as oppressive (Watson 2019). Labels can be seen as part of a toxic contagion, where harmful ideas and practices spread along social networks like a virus (Lavis and Winter 2020). But in queer activism, labels are seen as having emancipatory potential (e.g., Warner 1991). Queer labels have been at the centre of an extraordinarily successful political mobilisation, creating communities of interest in moments of what Gayatri Spivak calls ‘strategic essentialism’ whilst offering the conceptual resources for self-understanding, self-exploration and sociality (Spivak 1988). Queer labels seem like the antidote to repression, or ignorance, or shame. There is some crossover with mental health labels in the arena of campaigning and advocacy. Anthropologist Bridget Bradley notes that individuals who come together to actively create a biosocial community, are united by, and motivated by, mental health labels (Bradley 2021). She stresses that community can develop into activism (which she dubs ‘biosolidarity’) such that biomedical labels can be a necessary ingredient of radical action.

In our ethnographic work, mental health labels and queer labels mix and merge. They no longer seem so different. In their social context, labels emerge as responses to a desire for specification. Some of the students we worked with appeared uneasy at the prospect of important areas of life being unclassified or uncategorised. They seemed to have prospective concerns for what philosopher Miranda Fricker calls ‘hermeneutical injustice’ that is, of important areas of experience being inexpressible or impossible to understand or communicate (Fricker 2007). Fricker warns that hermeneutical injustice can leave a person ‘deeply troubled, confused and isolated’ (ibid:151). If key experiences appear to fall off the map, they are lost, even erased. Labels may be an antidote. They constitute a means of recognition and a way of ensuring that life remains comprehensible and communicable.

Our ethnographic data reveal a wide range of different relationships between label and person, including how seriously students take labels, when and to what degree they are sincere or cautious or doubting or even dissenting. One student told us:

“I once participated in a stand-up comedy course, the culmination of which was a live showcase to a large audience. Throughout, the teachers put great emphasis on the need for us to develop our stage persona; what is about ourselves that makes us unique?; which of our weird and wonderful traits, accents, appearances or deformities could we ridicule in order to endear ourselves to the crowd? After several weeks of self-examination, I conceived the persona that would allow me to talk and behave authentically on stage. I walked awkwardly to the microphone, resisting any eye contact with the audience, and made my introduction; ‘Hello, I’m ….. I’m often mistaken for being German, or Danish or……...austistic’.”

Here the fluid, flexible and playful are central to the social life of labels.

As a student, the words “mental breakdown” are often used without further thought of their origins. One interlocutor told us:

“There are countless times where I have heard friends, or myself, the night before a deadline “having a total breakdown” or with “anxiety going through the roof right now”.

These expressions, while stemming from a stress-induced place, purposefully exaggerate the situation. This is particularly frequent online. When someone else can’t see how stressed you are, it is easier for them to believe the extent of your distress using terminology akin to the extremes experienced in mental illness. This exaggeration becomes almost comical, a way of simultaneously both adding and reducing the to the scale of the problem. Humorous exaggeration can thus be a way of expressing emotion without raising alarm. Students learn to fine-tune their use of labels to harness their potential advantages while still preventing stigma.

The use of humour including mental illness disclosure is a polarizing topic. Previous psychology research has reported the benefits of humor in reducing stigma and facilitating conversations about illness that would be taboo in other contexts. Jensen’s study (2018) illustrated how joking about mental illness in a homeless shelter allowed staff and service users to have frank discussions about these stigmatizing traits. He described this as “when there are no inappropriate things to joke about, there are also no inappropriate important issues to discuss.” (ibid:32). Openly joking about stigmatizing traits, including illness, queer identities, and homelessness, reduced the negative valence of the attached emotions, reducing stigma and enabling nuanced conversations about their experiences. On the other hand, individuals with a mental illness diagnosis have shared their disagreement with undiagnosed individuals using this kind of humor. A blogpost from BP Hope, an online community that aims to raise awareness and support for bipolar disorder, highlights the importance of being educated on mental illness before engaging in jokes about it. While these can be therapeutic, the respect and dignity of those affected have to be considered. Jokes about suicide are often triggering, and describing others’ negative traits using a mental health label adds to the stigma surrounding the conditions (Paquette 2021). Similarly, an online forum from the Mood Disorders Society of Canada (2017) discusses how jokes about depression promote the idea that depression is a personal choice, which negates their experiences of struggle. As such, this type of humour can be conducive to both connection and disconnection, where those who have a mental illness are able to bond over it and destigmatize experiences, while use outside of this context may contribute to stigma.

The examples described depict students who are flexible, but careful, in their use of mental health labels. Contrary to previous theories of stigma, the humorous way in which these labels are sometimes delivered is empowering for students, relating to social bonding and coping, rather than alienation and helplessness. These instances move beyond a purely medical application of labels, towards a less boundaried social definition.

## Relational Power of Labels: Desirability and Validation in Social Contexts

Student interactions are increasingly digital, with social media providing a distinctive forum for relationship building. The social life of mental health labels online represents a further extension of their properties as technologies that can be used flexibly to achieve social desirability. Dark humour and meme culture propagated through these platforms is a thriving form of communication among students. But there are wider uses relating to social validation and identity building that permeate into students’ lives. Online pages, such as @the.sad.lines and @sadnesslosthim on Instagram, posting ‘beautifully tragic’ quotes and images are commonly shared by many young people, depicting deeper sensibilities and mysteries that can be strategically used to create a more alluring persona. This has given rise to the ‘sad boy’, ‘soft boy’ or ‘e boy’ archetype in the dating scene, referring to a young guy who purposely presents as ‘depressed’ or ‘socially anxious’ as a mechanism to attract others who feel a need to comfort or change them. A soft boy might present a misunderstood persona on social media linking dark quotes from music and literature, including various references to substance misuse and often keeping others’ attention through using their destructive behaviours to make them unpredictable and distant. The Tab, which is a popular student-written publication in the UK, as well as other publications such as Metro and Vice have reported on this archetype’s traits (Erskine 2019; Lindsay 2019 Shadijanova, 2019). The Gen-Z subtype identified, the ‘E-boy’, is described as “performatively sad” (Bassi 2019).

The most popular report of this has been the instagram page @beam_me_up_softboi, where people share screenshots of these type of interactions (beam_me_up_softboi 2022). In these, it is evident that presenting behaviours and language associated with mental illness is something to be romanticized. It can provide a certain “bad boy” magnetism, or appeal to what participants hope is an intrinsic human need to nurture:
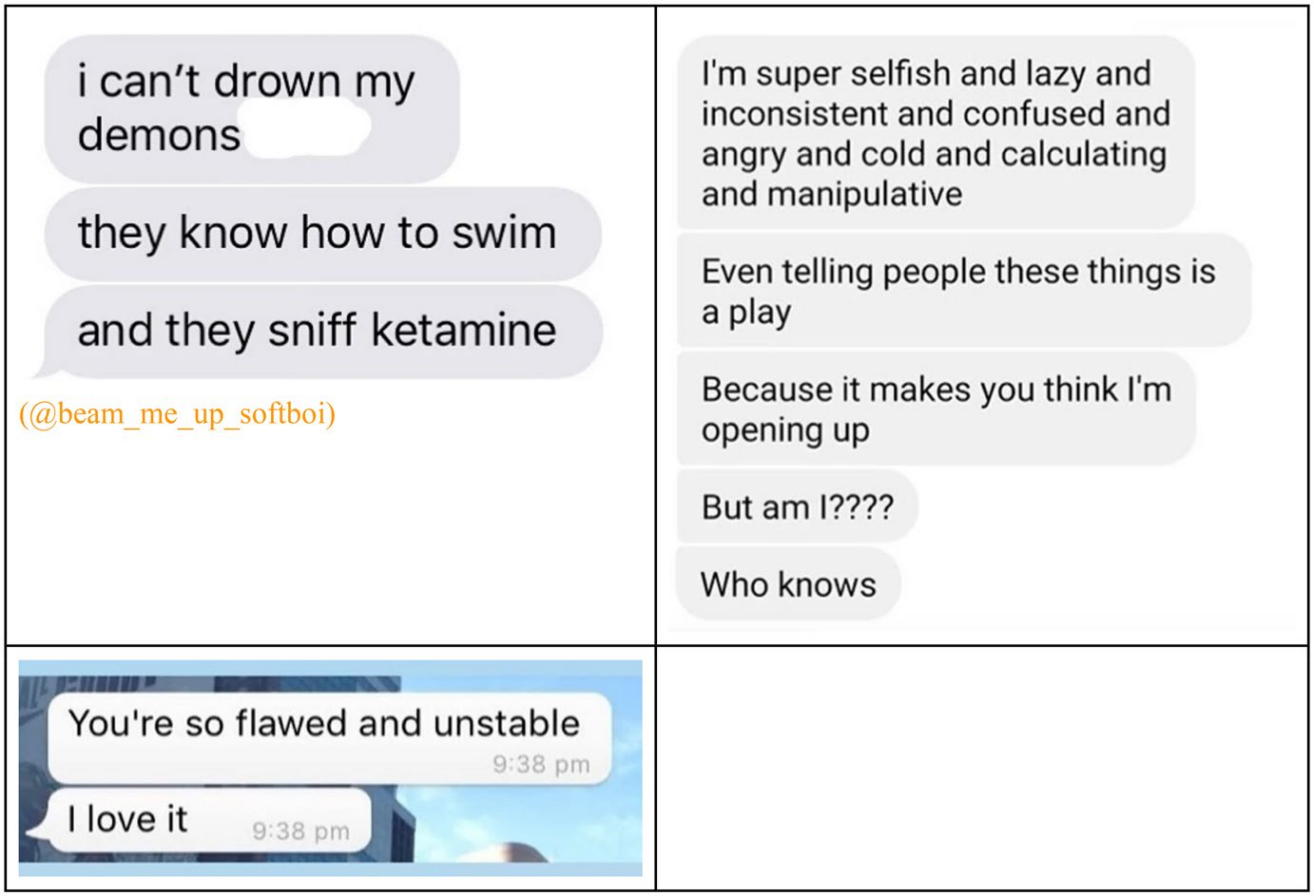


Students told us this is a common presentation in the dating scene, primarily among men. This might be the point in our ethnographic material where mental health labelling most sharply diverges from a Goffmanesque account of reputational damage leading to competitive disadvantage on the social stage. One student described the role of social media in enabling the use of mental health labels in relationships:

“I think generally our generation has a massive influence on social media as well. Our generation just jokes about suicide a lot. I think we’ve normalised it a lot within our discussions. I was reading this thing about tumblr and other social media and how it romanticizes depression and all these mental health things that make us want to fit the aesthetic.”

It’s not that this social stage is not competitive, it’s that categories that in other contexts might be stigmatising, here bring competitive advantage.

These labels are not a source of alienation or shame, but are valued by some students because they provide transparency and bonding in relationships when disclosed with careful consideration. Some students expressed scepticism about this kind of self presentation, but others said that disclosing mental illness allows them to be open with a potential partner and build a safer environment from the beginning. They can provide an explanation for certain behaviours and beliefs, such as reactions to specific triggers or experiencing periods of low mood, wrapped in a single concept that can promote better understanding and care. However, we were told that the use of labels to create relational allure is less effective on campus than it had been at school.

These findings do not suggest that mental illness stigma has completely disappeared, but instead may have shifted into a more covert self-stigma where the experiences and vulnerability underlying the labels are still unpalatable in a social conversation. Our interlocutors stated that they knew how much to disclose, and, like Clare, could predict the impact that mental health language would have without having to provide further detail.

## If Mental Health Labels have a Social Life, What does this Mean for Researchers?

If we see students who are active and agential in their dealing with mental health labels, this might have consequences for research methodologies. For example, previous research has shown that patient reported mental health does not accurately reflect clinician assessment. Eaton and colleagues describe the issue of weak agreement between self-reported instruments and clinician conducted diagnostic interviews as threatening to the credibility of prevalence estimates for specific disorders (Eaton et al. 2000). Their research found that individuals had a tendency to underreport symptoms in major depressive disorder, primarily those attributed to life-crises and medical conditions. One way to interpret this is as a failure on the part of the patient, such that if they had a better sense of what depression was, they might be able to report their state more accurately. Our findings suggest something different. It may be that students are simply responding to mental health labels as boundary objects that are legible in a range of domains. They are not restricted to a strictly biomedical reading because the terms themselves have a wider social life in which their meaning undergoes changes. In this interpretation, the problem of underreporting or over-reporting symptoms becomes a methodological failure, in that researchers need to accommodate the social life of medical labels.

Student mental health is increasingly being measured at national levels to gain a temperature check of this population’s experience over the years. Organisations such as the Office for National Statistics (ONS) and the Higher Education Policy Unit (HEPI) have reported on student mental health and wellbeing using quantitative survey measures, both finding higher levels of anxiety in the student population than the general population (Johnston 2021; Neves and Hewitt 2021). These are understood to provide valuable insight into the broader trends in the student experience with implications for policy and practice. However, the interpretation of this data is not straightforward. Where the HEPI survey measures anxiety as a ten-point scale in regards to “how anxious did you feel yesterday”, this measure does not acknowledge the way students understand and use mental health language, providing a potentially misleading account of the medical construct of anxiety. On the other hand, the ONS measures anxiety using two items of a validated tool, the GAD-7, which focus on specific symptoms such as “worrying” or “feeling nervous.” While a symptom focused approach to measurement begins to provide a more nuanced depiction of the student experience, universities may find that it does not reflect the number of students reporting anxiety to access support services, as we have seen with our interlocutors that diagnostic language often facilitates receiving support. We believe that the data from these examples should be interpreted with caution, as students may be interacting with the items in the surveys rather differently than envisaged by the researchers.

Similarly, some research assumes a fairly direct link between label and supposed explanatory models or beliefs about aetiology. Samouilhan and Seabi (2010) found that aetiological beliefs impact help-seeking styles, where beliefs that a mental disorder is caused by biology result in individuals seeking medical help, whereas stress-related causes are approached through social support. Again, our work suggests a degree of caution is needed. It appears that individual students engage with mental health labels without necessarily being committed to the implicit medical model that may lie behind the label. In certain settings a medical label is advantageous. This cannot be assumed to indicate a deep level of consensus.

Within clinical psychology, service user research and activism and critical currents within psychiatry itself, caution and even scepticism towards labelling and the ‘medical model’ is widespread. Clinical psychologist Lucy Jonstone argues: ‘it is no longer scientifically, professionally or ethically justifiable to insist on psychiatric diagnosis as the only way of describing people’s distress and to deny people the opportunity to explore alternatives’ (Johnston 2021:13). Our work suggests that labelling is ambiguous and flexible, such that even if a medical professional insists on a psychiatric diagnosis as Johnstone describes, that does not prevent a person from exploring alternatives. Students engage with multiple labels and perspectives on distress, although such an exploration may occur away from the clinical gaze.

## Discussion

In her discussion of the way Biomedical terms like ‘PTSD’ and ‘depression’ enter Iranain society, Orkideh Behrouzan finds the notion of medicalisation to be reductive (Behrouzan 2016). In a way that might be familiar to readers of this paper, she describes how labels are not fixed and do not discursively colonise Iranians. Rather, they become a kind of ‘cultural resource and generative process of meaning making’ that makes possible ‘new cultural forms’ (ibid:35). We want to make a similar argument. In the opening vignette, we saw that Brian had a complex, multifaceted relationship with the bipolar label. For contemporary students, mental health labels make possible forms of identity, humour and agency that otherwise wouldn’t exist. They are campus technologies. Ultimately, the experiences of our interlocutors demonstrate the flexibility of labels in the social world, holding the power to make a student legible to an institution, and render distress intelligible, valid, manageable, and sometimes socially helpful. Labels can create romantic allure, promote bonding, and help with self definition and by means of specification, domesticate otherwise unpredictable and unruly social and experiential worlds. The belief that disclosing mental illness is always met with negative judgment and social harm may no longer be correct. In a society where the use of all kinds of labels prevails in various aspects of life, labelling might need to be retheorised.

When we began this project, we gained a sense that mental health labels could not simply be seen as harmful, or dominating, or stigmatising and that students are active labellers as much as they are labelled. Our ethnographic work helped develop this intuition. The students we spoke with are self-aware consumers of labels. They were happy to describe how they had learned to use labels in focussed and fine-tuned ways to make their needs and preferences legible to the university and to carve out social identities, patterns of sociality and self-understanding. They are neither resisting biomedical hegemony nor dominated by it. The adoption of medical labels does not necessarily indicate that people medicalise their problems, or internalise medical models, or are subjectified; the students we got to know are not passive recipients of biopower. Rather they draw on biomedical and other labels to weave together complex, heterogeneous and flexible understandings of themselves, their distress and its entitlements. Labels that appear to be oriented towards making sense of experiences and promote better mental health, might be employed to negotiate friendships, bond over deadline stress, or develop an online persona.

Ideas that seem commonplace or obvious to the students we worked with were surprising and unexpected to researchers. We cannot be sure that our findings apply across all student populations in the UK. Various dynamics, such as class or racial privilege may play a role. Further investigation is required. But one reason why there is such a limited trace of this in published research is archaeological, having to do with core theoretical assumptions that have become embedded in social science literature. These assumptions are perpetuated methodologically. This points to the potential contribution of coproduced ethnography in mental health research. When used in bureaucratic or healthcare contexts, labels can look like formal descriptors of fixed inner states. That is part of their rhetorical power. But rather than take this at face value, we need to view with caution research methodologies that replicate closed bureaucratic thinking. The openness and flexibility of collaborative ethnographic working enables an attentiveness where other methods, whatever their advantages, can be tone-deaf, even tin-eared. This suggests a limitation to research methods or forms of knowledge based on hypotheses or fixed investigative trajectories. Less predictable, more spontaneous (and so less immediately fundable) forms of research might play a key role in understanding student mental health.
